# Extrathymic Autoimmune Regulator (AIRE) expression in rheumatoid arthritis

**DOI:** 10.1186/1479-5876-10-S3-P33

**Published:** 2012-11-28

**Authors:** Ae-Ri Noort, Katinka PM van Zoest, M Christina Lebre, Paul-Peter Tak, Sander W Tas

**Affiliations:** 1 Division of Clinical Immunology & Rheumatology, AMC, University of Amsterdam, the Netherlands

## Background

The Autoimmune Regulator (AIRE) is a transcription factor that is involved in the negative selection of self-reactive thymocytes in the thymus and is pivotal in the establishment of central tolerance. Recently, AIRE protein has also been detected in peripheral lymphoid organs, predominantly in dendritic cells (DC). In these peripheral sites, AIRE was found to regulate the expression of a group of tissue-specific antigens, suggesting that peripheral AIRE may play a complementary role in tolerance induction. It is currently unknown whether AIRE may play a role in inflamed tissues associated with ectopic lymphoid neogenesis, such as rheumatoid arthritis (RA) synovial tissue (ST).

## Objective

To document and further characterize extrathymic AIRE expressing cells in ST and paired peripheral blood (PB) mononuclear cells (MCs) as well synovial fluid (SF) MCs of RA patients.

## Methods

ST was obtained via mini-arthroscopy from inflamed joints of RA or undifferentiated arthritis (UA) patients.

Expression of AIRE was evaluated using immunohistochemistry and immunofluorescence (IF) microscopy.

AIRE expression was also investigated in PB and SF DC using flowcytometry.

## Results

AIRE expressing cells were detected in 80% of RA ST and in contrast only in 25% of UA ST. Further characterization using IF microscopy revealed that these cells were predominantly CD1c (BDCA1)^+^ myeloid (m)DC. Interestingly, a significantly higher percentage of CD1c+ mDC in RA SF expressed AIRE (55 + 5 %; n=12) compared to RA PB (20 + 3 %; n=12; p<0.05) and healthy PB (19.7 + 2 %; n=5; p<0.05).

## Conclusions

Extrathymic AIRE expressing cells are present in RA ST and RA SF, suggesting a role in synovial inflammation. These AIRE expressing cells appear to be mainly CD1c+ mDCs. Extrathymic AIRE expression in RA may be an attempt to control inflammation through the induction of peripheral tolerance to antigens involved in the perpetuation of the chronic inflammatory response. This mechanism may be exploited to develop new treatments for RA patients.

